# Detection of gases and organic vapors by cellulose-based sensors

**DOI:** 10.1007/s00216-023-04649-z

**Published:** 2023-03-31

**Authors:** Francisco Pena-Pereira, Isela Lavilla, Inmaculada de la Calle, Vanesa Romero, Carlos Bendicho

**Affiliations:** Centro de Investigación Mariña, Departamento de Química Analítica e alimentaria, Grupo QA2, Edificio CC Experimentais, Universidade de Vigo, Campus de Vigo, As Lagoas, Marcosende, 36310 Vigo, Spain

**Keywords:** Microfluidics, Cellulose and nanocellulose, Paper-based analytical devices, Sensing, Vapor generation, Volatile compounds

## Abstract

The growing interest in the development of cost-effective, straightforward, and rapid analytical systems has found cellulose-based materials, including cellulose derivatives, cellulose-based gels, nanocellulosic materials, and the corresponding (nano)cellulose-based composites, to be valuable platforms for sensor development. The present work presents recent advances in the development of cellulose-based sensors for the determination of volatile analytes and derivatives of analytical relevance. In particular, strategies described in the literature for the fabrication and modification of cellulose-based substrates with responsive materials are summarized. In addition, selected contributions reported in the field of paper-based volatile sensors are discussed, with a particular emphasis on quick response (QR) code paper-based platforms, intelligent films for food freshness monitoring, and sensor arrays for volatile discrimination purposes. Furthermore, analytical strategies devised for the determination of ionic species by in situ generation of volatile derivatives in both paper-based analytical devices (PADs) and microfluidic PADs will also be described.

**Graphical abstract:**

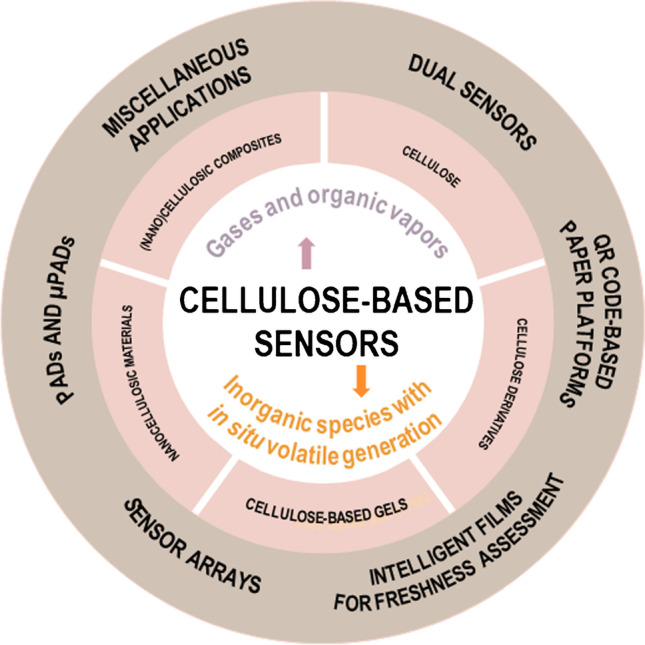

## Introduction

Cellulose has been profusely used for analytical purposes since the introduction of spot tests in the mid-nineteenth century [1]. Cellulose and cellulose-derived materials show remarkable properties, including wide availability, biodegradability, biocompatibility, inexpensiveness, or disposability. In addition, these bio-based polymers are compatible with a wide range of chemicals, allowing them to be stored and transported. In recent years, there has been a resurgence of interest in this topic following outstanding contributions of Whitesides’ group to the fabrication of paper-based analytical devices (PADs) including microfluidic PADs (µPADs) and, especially, to their combined use with non-conventional detection systems [2, 3]. These inspiring works, identified as paradigmatic examples of “frugal science” [4], have led to an impressively rapid development of analytical strategies of growing acceptance in scientific and technological fields.

The abovementioned properties of cellulose are particularly relevant for the development of paper-based devices with analytical capabilities. Furthermore, the white color of cellulose substrates is particularly advantageous for contrast enhancement when colorimetric readout is used, whereas the possibility to obtain alternative cellulose-based materials with remarkable transparency expands even more their applicability. Besides, the porous characteristics of cellulose substrates confer additional benefits, including the spontaneous capillary action and the efficient interaction of analytes with the corresponding immobilized recognition elements, thus leading to relatively rapid responses. These features make them unrivaled substrate candidates for the development of analytical devices with potential applicability in chemical analysis and diagnosis. It should be highlighted that PADs fulfil the requirements to meet the ASSURED (Affordable, Sensitive, Specific, User-friendly, Rapid and robust, Equipment-free, and Deliverable to end users) criteria in the area of diagnostics for the developing world [5].

**Scheme 1:**
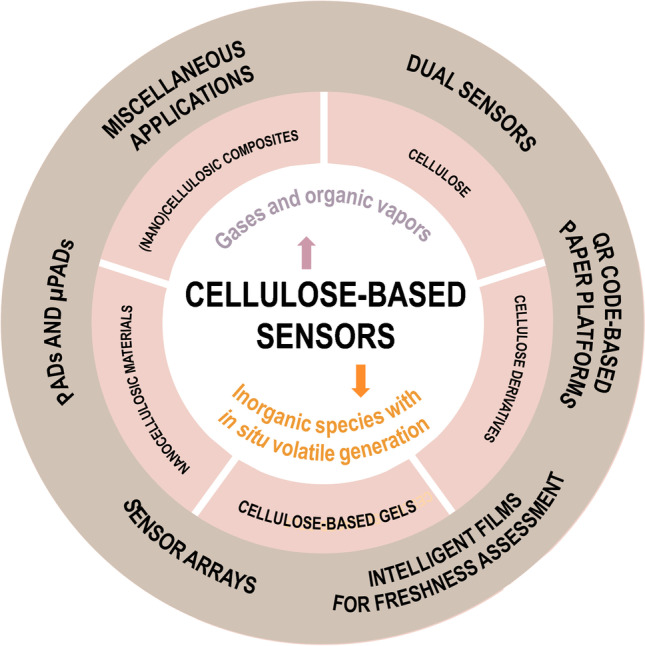
Schematic representation of the contents discussed in this work

The interest in the topic is evidenced by an increasing number of original contributions and excellent review articles dealing with cellulose-derived materials [6–10], including their fabrication and general applications and, particularly, both general and specific aspects of PADs [11–14]. However, as far as we are aware, reviews focused on recent contributions devoted to the detection of gases and organic vapors involving cellulose-based substrates are missing. Thus, the scope of this review, depicted in Scheme 1, focuses on recently reported cellulose-based sensors for volatile sensing. An overview of cellulose-related materials employed for the preparation of volatile sensors, including cellulose, cellulose derivatives (e.g., cellulose acetate), cellulose-based gels (e.g., hydrogels, xerogels or aerogels), nanocellulosic materials (namely, bacterial cellulose, cellulose nanofibrils, and cellulose nanocrystals), and (nano)cellulose-based composites, is first provided. Fabrication and modification strategies reported for the development of cellulose-based sensors are subsequently described. Selected contributions involving single and dual readout approaches for the detection of gases and organic vapors are discussed, paying special attention to sensor arrays for volatile discrimination purposes, or the application of cellulose substrates in intelligent films for the monitoring of food freshness. Furthermore, sensing strategies developed for the determination of ionic species following in situ generation of volatile compounds with both paper-based spot tests and microfluidic devices are highlighted. Special attention is devoted to the design and fabrication of paper-based sensors, the recognition elements used, and the chemistry behind the sensing strategies. Furthermore, technological advances and remarkable contributions are highlighted, and challenging aspects to be considered in the development of cellulose-based sensors are finally outlined.

## Cellulose and their composites in vapor sensing

Cellulose is a highly abundant bio-based polymer whose structure is based on linear chains of β-1,4-linked glucose units. It is mainly present in wood and cotton, but also found in marine animals, algae, bacteria, fungi, and even amoeba [7]. Cellulose shows excellent characteristics, including biocompatibility and biodegradability, sustainable production, and mechanical robustness [8]. The physicochemical properties of cellulose can be tuned by chemical modification of the abundant hydroxyl groups [6, 15], thus leading to derivatives with either hydrophilic or hydrophobic features.

A wide range of cellulose materials have been employed for the preparation of devices for volatile sensing. Commercial filter paper of different grades is by far the most common cellulose substrate used with this aim. Chromatographic paper, printing paper, and cardboard substrates have also been employed. The wide and ready availability of the abovementioned substrates, together with their affordability, has facilitated the evaluation of novel responsive materials acting as chemosensors for volatile sensing purposes. Even though cellulose-derived materials are commonly used as scaffolds in many sensing approaches, they have also shown more active roles in the development of sensors for gases and organic vapors.

Cellulose derivatives have also been used to a significant extent, including the fabrication of organic–inorganic hybrid gas sensor membranes [16] and responsive coating materials for the detection of volatiles [17]. Cellulose acetate shows excellent film-forming ability, and its thermoplastic characteristics have also enabled the fabrication of films and biodegradable packaging bags modified for the detection of volatiles [18]. In addition, cellulose derivatives have been used for the fabrication of gels. For example, carboxymethyl cellulose enabled the fabrication of a flexible Au nanoparticle (NP)-modified MXene hydrogel that exhibited antifreezing properties [19]. Cellulose derivatives have also been employed for the fabrication of microporous vapochromic xerogels loaded with pH-responsive materials [20]. In addition, a carboxylated cellulose aerogel [21] and a cellulose/reduced graphene oxide composite aerogel [22] have been reported as sensitizing materials for quartz crystal microbalance (QCM) vapor sensing.

In recent years, nanocellulosic materials are receiving increasing attention. Three categories of cellulose nanomaterials have been identified by the International Organization for Standardization, including bacterial cellulose, cellulose nanofibrils, and cellulose nanocrystals, all of them characterized by showing at least one dimension at the nanoscale [23]. Bacterial cellulose, produced with particular efficiency by the *Gluconoacetobacter xylinus* family [6, 8] and composed by cellulose fibrils, is characterized by its high purity and absence of hemicellulose or lignin [7]. Cellulose nanofibrils usually show a diameter in the range of 5 to 60 nm and a length lower than 10 µm, whereas cellulose nanocrystals show lower aspect ratios but higher crystallinity than cellulose nanofibrils [8]. Nanocellulose shows remarkable properties when compared with conventional paper, including optical transparency, low surface roughness, and mechanical stability [10].

Bacterial cellulose has been employed for the preparation of flexible and transparent plasmonic nanopapers [24]. It has also been employed for the preparation of hybrid materials, including ionogels, i.e., ionic liquid-based hybrid materials, modified with fluorescent probes [25] and hybrid nanocomposites [26].

Cellulose nanofibers have also facilitated the fabrication of composites aimed at gas sensing with improved performance. For instance, composites based on cellulose nanofibers and carbon nanotubes [27] and graphene [28] have been reported for humidity sensing due to their hygroscopic nature. Furthermore, a sulfonic acid–cofunctionalized nanocellulose/graphene oxide membrane has been applied in an electrochemical gas sensor [29]. The properties of cellulose nanofibers allow enhancing of dispersion of carbon nanomaterials [30, 31], a fact of paramount importance to obtain stable and uniform printable inks for the fabrication of sensors [30]. Thus, the formation of cellulose nanofiber/carbon black [30] and cellulose nanofiber/graphene nanoplatelet [31] composite inks with a uniform dispersion of carbon-based nanomaterials for printed flexible sensors has been reported. Additionally, cellulose nanofibers have played a role as nucleation templates in the fabrication of nanocomposites [32]. Transparent films of cellulose nanofibers have also shown remarkable properties for the development of gas sensors with optical readout [33].

Cellulose and cellulose-derived nanocrystals have been employed as reinforcing agents for film production and volatile sensing [34, 35]. In addition, cellulose nanocrystals have shown a remarkable role as stabilizers of in situ formed NPs being exploited for sensing volatiles [36]. Interestingly, cellulose nanocrystal–based chiral nematic materials can be formed by evaporation-induced self-assembly, and some captivating contributions on the responsiveness of iridescent cellulose nanocrystal films for gas sensing have been reported [37, 38].

## Fabrication of cellulose-based sensors

**Fig. 1:**
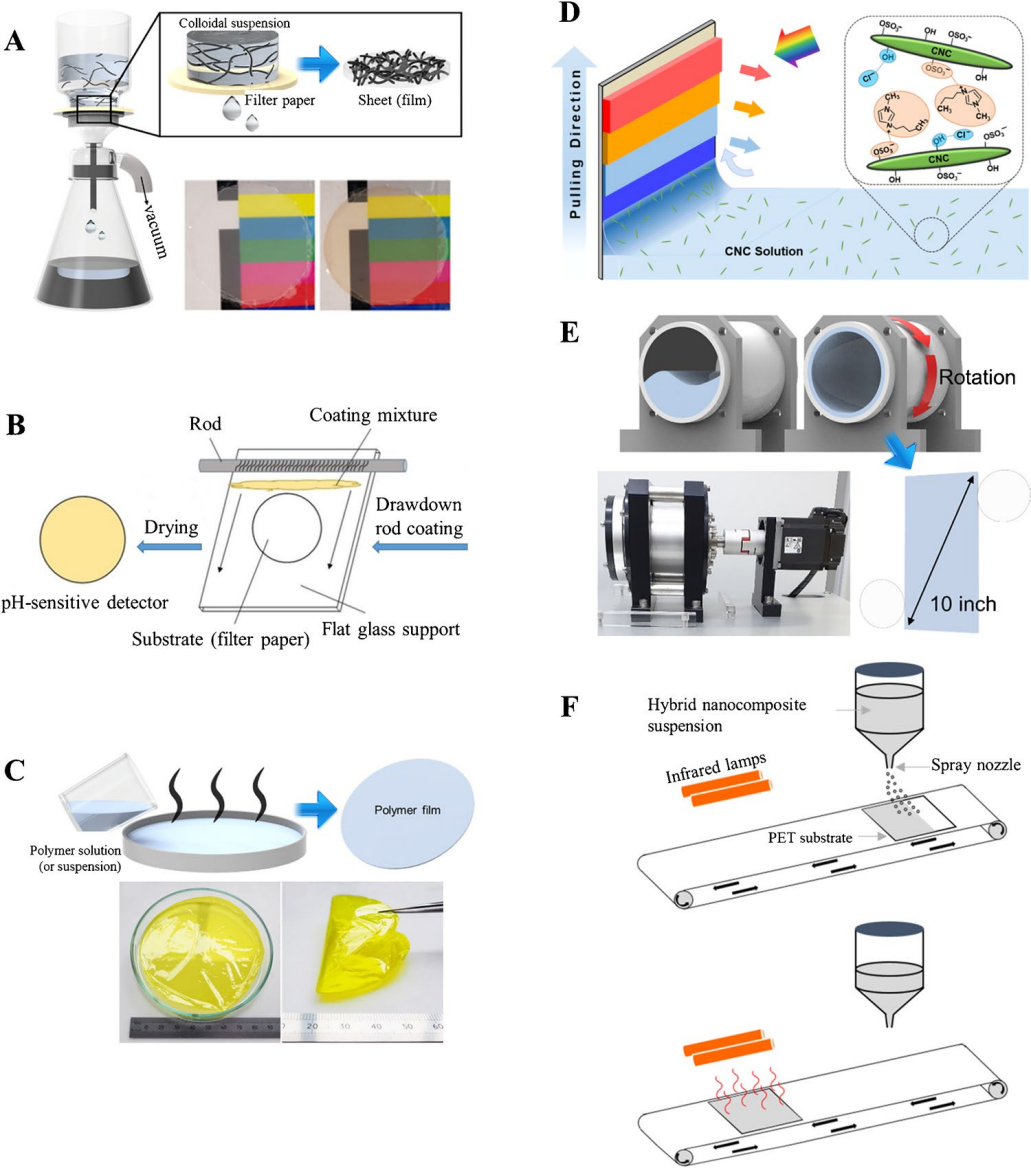
Selected techniques employed for the fabrication of cellulose-based sensors. Vacuum filtration (**A**), drawdown rod coating (**B**), solvent casting (**C**), dip-and-pull (**D**), horizontal centrifugal casting (**E**), and thermal spray (**F**). Reprinted with permission from [26, 42, 54, 55]

The immobilization of responsive materials in cellulose substrates is commonly performed by dip coating, drop casting, and vacuum filtration techniques. Obviously, significant physical interactions or chemical linkages between the responsive material and the cellulosic substrate are required to ensure the immobilization of the sensing material. Thus, previous modification of the substrate might be required for improving retention. The dip-coating technique is carried out by the immersion of cellulose substrates in a solution containing the recognition element for a prescribed time, followed by solvent evaporation. Drop casting is also commonly used. It generally involves the deposition of a highly reduced volume (e.g., 1–10 µL) of solution on the cellulose substrate which, however, can lead to a less homogeneously distributed responsive material due to the concentration gradient associated to the capillary-driven flow on the cellulose substrate. Vacuum filtration (Fig. 1A) has enabled a variety of composites to be deposited on cellulose-based substrates [28, 39, 40] or alternative polymeric supports [41]. Alternative film-forming techniques have been proposed, allowing for a higher control on the areas of the cellulose substrate to be modified, as well as on the amounts of solutions required for the preparation of sensors. For example, a conventional drawdown rod coating method (Fig. 1B) has been used to deposit coating materials with a pH-responsive dye on filter paper [42]. In addition, a number of paper-based sensors have been fabricated by handwriting. For instance, oxidized-multiwalled carbon nanotube (o-MWCNT) ink markers [43] or refillable pens or cotton swabs dipped in the sensing materials [44] have been used. Furthermore, both inkjet printing [45, 46] and screen printing [30, 47–49] allow the formation of uniform and homogeneous sensing areas in cellulose substrates with careful control of the areas to be printed. In any case, the compatibility of responsive compounds with the rest of the chemicals required to obtain printable materials must be ensured. Alternatively, the formation of hydrophobic barriers in cellulose substrates (e.g., by wax printing), widely used in paper-based analytical devices, simplifies the modification of specific areas of the substrate (e.g., reaction and/or detection areas) with a given amount of responsive materials, mainly by drop casting [50–52]. Additional information on the fabrication strategies of PADs and µPADs can be found in excellent reviews [11, 12, 14, 53].

A number of contributions involve the formation of cellulose-based films or the deposition of cellulose-containing responsive materials onto other solid substrates. In this respect, solvent casting (Fig. 1C) is widely used for obtaining films of polymers, this strategy being also applicable to cellulose-based materials. However, the achievement of uniform and thin films is not guaranteed with this technique. Different alternatives have been explored to deposit films of cellulose-derived materials on solid substrates with a controllable thickness. A dip-and-pull process (Fig. 1D) involving a vertically positioned syringe pump has enabled the deposition of thin coating films of cellulose-based materials onto solid substrates [54]. This process has been exploited for the preparation of cellulose nanocrystal films on silicon substrates, through evaporation-induced self-assembly, thus achieving thicknesses in the range of 100–300 nm. Recently, the horizontal centrifugal casting technique (Fig. 1E), based on the combined use of centrifugal force and horizontal arrangement of a cylindrical mold [55], has been reported and applied to the preparation of transparent nanocellulose-based gas sensors [33]. Thermal spray (Fig. 1F) has also been used to obtain thin films of responsive materials (e.g., hybrid nanocomposites [26]) over solid substrates. Among other features, the thermal spray technique allows the formation of optically transparent films with thicknesses even lower than 1 µm [26].

## Cellulose-based sensing of gases and organic vapors

In this section, recent contributions on cellulose-based sensors for detecting either inorganic or organic volatile compounds are described and discussed. Tables 1 and 2 provide information of interest for the cellulose-based sensors discussed in this section. Selected cellulose-based sensors involving single readout approaches are firstly described, followed by dual-mode detection strategies (Table 1). Finally, the section deals with cellulose-based sensors that possess an ever-increasing impact, namely cellulose-based sensors for smart food packaging and paper-based optoelectronic noses (Table 2).

**Table 1:** Selected applications of cellulose-based sensors for detection of gases and organic vapors involving single and dual readout approaches

Analytes	Recognition element	Cellulose material	Detection	LOD	Analysis time	Ref
Diethyl chlorophosphate	HEMBAO	Cellulose nanofibers	Colorimetric	10.38 µM	–	[33]
Aldehydes	IL-cellulose nanocrystals	Cellulose nanocrystals	Colorimetric	ca. 100 ppm (naked eye), 0.5 ppm (RGB)	–	[54]
RH and formaldehyde	Cellulose nanocrystals	Cellulose nanocrystals	Colorimetric	≤43% (RH); ≤0.14 mg/m^3^ (formaldehyde)	–	[37]
Indole	DMACA	Chromatography paper (grade 1)—QR code paper	Colorimetric	1 ppm	10 min	[50]
RH, CO_2_, NH_3_, H_2_S	Nafion-crystal violet (RH), α-naphtholphthalein (CO_2_), bromophenol blue (NH_3_), Cu-PAN (H_2_S)	Paper—QR code paper	Colorimetric	44.6–46.7% (RH); 0.7% (CO_2_); 0.6–0.7 ppm (NH_3_); 0.01–0.13 ppm (H_2_S)	2 min	[48]
H_2_S	Bimetallic europium(III)/copper(II) complex chemosensor	Filter paper	Fluorimetric	100 ppb	0.5 min	[57]
H_2_S	Bis(2-aminobenzoic)palladium(II)	Filter paper	Fluorimetric	2 ppb	15 min	[58]
O_2_ and CO_2_	PtTFPP and HPTS	Ethyl acetate	Fluorimetric	<20%(v/v)	Response/recovery times: 15/41 s (O_2_) and 7/39 s (CO_2_)	[59]
CO_2_	Np-P4VB	Filter paper	Fluorimetric (ratiometric)	5.7 ppm	Response/recovery times: 100/45 s	[60]
H_2_S	Cu-PDA/luminol-H_2_O_2_	Chromatography paper	Chemiluminescence	0.8 ppb and ≤20 ppb	2 min	[61]
RH	o-MWCNTs	Printing paper, weighing paper, and cardboard	Electrical current change	≤33% RH	Response/recovery times: 5–8 min/7–11 min	[43]
RH	Cellulose nanocrystals/graphene	Cellulose nanocrystals	Electrical resistance change	≤15% RH	Response/recovery times: 45 s /33 s	[28]
RH	Nanofibrillated cellulose/carbon nanotubes	Nanofibrillated cellulose	Electrical current change	≤11% RH	Response/recovery times: 330 s /377 s	[41]
RH	Cellulose nanofibers/carbon black	Cellulose nanofibers	Electrical resistance change	≤30% RH	Response/recovery times: 10 s /6 s	[30]
RH	Cellulose nanofibers/graphene nanoplatelet	Cellulose nanofibers	Electrical resistance change	≤30% RH	Response/recovery times: 17 s /22 s	[31]
RH	Cellulose nanofibers/carbon nanotubes	Cellulose nanofibers	Electrical current change	≤11% RH	Response/recovery times: 322 s /442 s	[62]
RH	Nitrocellulose nanocrystals	Nitrocellulose nanocrystals	QCM	≤11% RH	Response/recovery times: 18 s /10 s	[35]
Acetone	Poly-4BCMU	Paper	Colorimetric/fluorimetric	–	–	[45]
NH_3_	Lead halide perovskites	Paper	Colorimetric/electrical resistance change	10–80 ppm/10–500 ppb	10– ≥240 s	[63]
SO_2_	rGO/MPy-AuNRs, anhydrous methanol, and starch-iodine complex	Filter paper	Colorimetric/SERS	1.45 and 5 µM/0.086 µM	8 min	[39]
Benzaldehyde	AuNRs-QDs@MOF	Filter paper	Fluorimetric/SERS	1.2 ppb/0.1 ppb	5 min	[40]
VSCs	Copper MOF	Carboxymethylated chromatography paper	Colorimetic/chemiluminescence	0.2 µM/8 nM	30 min	[64]

*4BCMU*, 5,7-dodecadiyne-1,12-diol bis[((butoxycarvbonyl)methyl)urethane]; *DMACA*, p-dimethylaminocinnamaldehyde; *HEMBAO*, ((E)-4-((E)-(4-((2-hydroxyethyl)(methyl)amino)phenyl)diazenyl)benzaldehyde oxime; *HPTS*, 1-hydroxy-3,6,8-pyrenetrisulfonic acid trisodium salt; *MOF*, metal-organic framework; *MPy*, 4-mercaptopyridine; *NRs*, nanorods; *Np-P4VB*, bis(4-pyridyl)-dineopentoxyl-*p*-phenylenedivinylene; *PAN*, 1-(2-pyridylazo)-2-naphtol; *PDA*, 1,10-phenanthroline-2,9-dicarboxylic acid; *PtTFPP*, platinum(II) meso-tetrakis(pentafluorophenyl)porphyrin; *QDs*, quantum dots; *rGO*, reduced graphene oxide; *RH*, relative humidity; *SERS*, surface-enhanced Raman scattering

**Table 2:** Selected applications of intelligent films and sensor arrays for detection of gases and organic vapors

Analytes	Recognition element	Cellulose material	Detection	LOD	Analysis time	Ref
H_2_S	Cellulose nanofiber–templated CuO decorated with W_2_S nanosheets	Cellulose nanofibers	Electrical resistance change	200 ppb	Response/recovery times: 37.2/33.9 s	[32]
TVB-N	Bromocresol purple	Whatman filter paper	Colorimetric	–	–	[42]
TVB-N	pH dyes based on ahthraquinone and azo chromophores (2)	A4 copy paper	Colorimetric	–	–	[49]
TVB-N	Bromophenol blue/bromocresol green mixtures	Filter paper	Colorimetric	≤50 ppm	–	[65]
TVB-N	Purple sweet potato (anthocyanins)	Filter paper	Colorimetric	–	–	[66]
TVB-N	TMB and TIF	Filter paper	Colorimetric	≤10 mg/L (TMA-N)	5 min	[67]
VOCs (ammonia, ethanol, methanol, toluene, etc.)	Nile red, methyl red, and Zn-TPP	Filter paper	Colorimetric	–	–	[56]
Acidic VOCs (acetic acid, lactic acid, etc.)	PAMAM dendritic macromolecules functionalized with spiropyran and doped with oxazolidine derivatives (4)	Filter paper	Fluorimetric	–	–	[68]
TVB-N	Gelatin/curcumin/chitosan microcapsules	Carboxymethyl cellulose-based films	Colorimetric	–	–	[69]
TVB-N	*Melastoma malabathricum* seed extract (anthocyanins)	Cellulose acetate and cellulose nanofibers	Colorimetric	1% NH_3_ (naked eye)	–	[18]
Aldehydes	Methyl red and NaOH	Filter paper	Colorimetric	–	–	[70]
Ethylene	Polydiacetylene and Lawesson’s reagent	Cellulose nanocrystals	Colorimetric	200 ppm (digital image, 600 ppm (naked eye)	24 h	[71]
RH	In situ grown Ag-MOFs	Carboxymethyl filter paper	Electrical resistance change	≤33% RH		[72]
Amines (3)	pH indicators (7)	Chromatography paper	Colorimetric	0.2–0.5 ppm	10 min	[76]
Phenols, alcohols, ketones, aldehydes, amines, acids, esters, arenes, and hydrocarbons (45)	AuNPs and AgNPs prepared using different capping agents (16)	PVDF paper	Colorimetric	8.72–23.83 ppb	90 min	[78]
Primary amines (7)	Dye-encapsulating polymer NPs with different polarity	A4 copy paper	Colorimetric	≤50 ppm	20 min	[46]
Nitroaromatics, aromatic aldehydes, arenes, alkyl alcohols, amines, and acids (30)	Soluble conjugated polymeric NPs (36)	Filter paper	Fluorimetric	7–65 ppm	30 min	[80]
Methanol, ethanol, ammonia, acetone, and toluene	Chemoresponsive dyes: pH indicators, metalloporphyrins and solvatochromic dyes (6) Chemiresistive materials: CNTs, PEDOT:PSS, graphite, and EMI TSFI	Chromatography paper	Colorimetric, electrical resistance change	≤7.5% (55–308 ppm)	30 min	[44]
Blood VOCs	AuNPs and AgNPs prepared using different capping agents (16)	PVDF paper	Colorimetric	–	270 min	[79]
Alcohols, ketones, ethers, esters, arenes and hydrocarbons, chlorinated solvents, ACN, DMF, and DMSO (15)	Benzothiazole-salicylidene derivatives (3)	Filter paper	Fluorimetric	≤2.5%(v/v)	5 min	[52]
Olive oil VOCs Nonanaldehyde	pH dyes, metalloporphyrins, solvatochromic dyes, and 2,4-dinitrophenylhydrazine (12)	Filter paper	Colorimetric	≤5 ppm	20 min	[51]
Alcohols, ketones, ethers, esters, arenes and hydrocarbons, chlorinated solvents, ACN, DMF, and DMSO (18)	Amphiphilic and bolaamphiphilic polydiacetylenes (8)	Chromatography paper	Colorimetric	–	60 min	[77]
Methanol, ethanol, acetone, and THF	Polymers of varying Hansen’s solubility parameters (8)	Chromatography paper	Degree of cantilever bending (naked-eye detection)	–	25 min	[81]

*ACN*, acetonitrile; *CNTs*, carbon nanotubes; *DMF*, dimethylformamide; *DMSO*, dimethyl sulfoxide; *EMI TSFI*, 1-ethyl-3-methylimidazolium bis(trifluoromethylsulfonyl)imide; *PAMAM*, poly(amidoamine); *PEDOT:PSS*, poly(3,4-ethylenedioxythiophene) polystyrene sulfonate; *PVDF*, polyvinylidene fluoride; *THF*, tetrahydrofuran; *TIF*, 2’,4’,5’,7’.tetraiodofluorescein; *TMA-N*, trimethylamine-nitrogen; *TMB*, 3,3',5,5'-tetramethylbenzidine; *TPP*, tetraphenylporphyrin; *TVB-N*, total volatile basic nitrogen; *VOCs*, volatile organic compounds

### Sensing of volatiles: single and dual readout approaches

Several cellulose-based sensors have been recently reported for the determination of volatile compounds with different signal readout approaches. Colorimetric detection, involving UV–vis spectrophotometry and, to a larger extent, smartphone-based devices and naked eye–based detection strategies, has been profusely employed for the detection of gases and organic vapors. Thus, the use of organic monomers and polymers with responsive features is considered in recent contributions for the colorimetric detection of gases and organic vapors. A transparent succinylated cellulose nanofiber film modified with (E)-4-((4-((2-hydroxyethyl)(methyl)amino)phenyl)diazenyl)benzaldehyde oxime has been used for the colorimetric detection of diethyl chlorophosphate (i.e., a nerve agent), in the vapor phase [33]. Remarkably, the reported film, compatible with a gas mask, shows promise for the detection of warfare agents without affecting the visible system.

The intriguing properties of iridescent materials based on cellulose nanocrystals have been assessed for the colorimetric detection of volatiles. For example, colored cellulose nanocrystal films functionalized with amine groups demonstrated their applicability for aldehyde detection [54]. Remarkably, the formation of multiple iridescent color bands (by controlling the pulling speed) enabled differentiating aldehydes (LOD: 0.5 ppm for formaldehyde) from non-aldehyde volatiles by principal component analysis (PCA). In addition, iridescent chiral nematic cellulose nanocrystal films and coatings have shown promising features for colorimetric humidity and formaldehyde gas sensing with remarkable reversibility (≥5 cycles) [37].

**Fig. 2:**
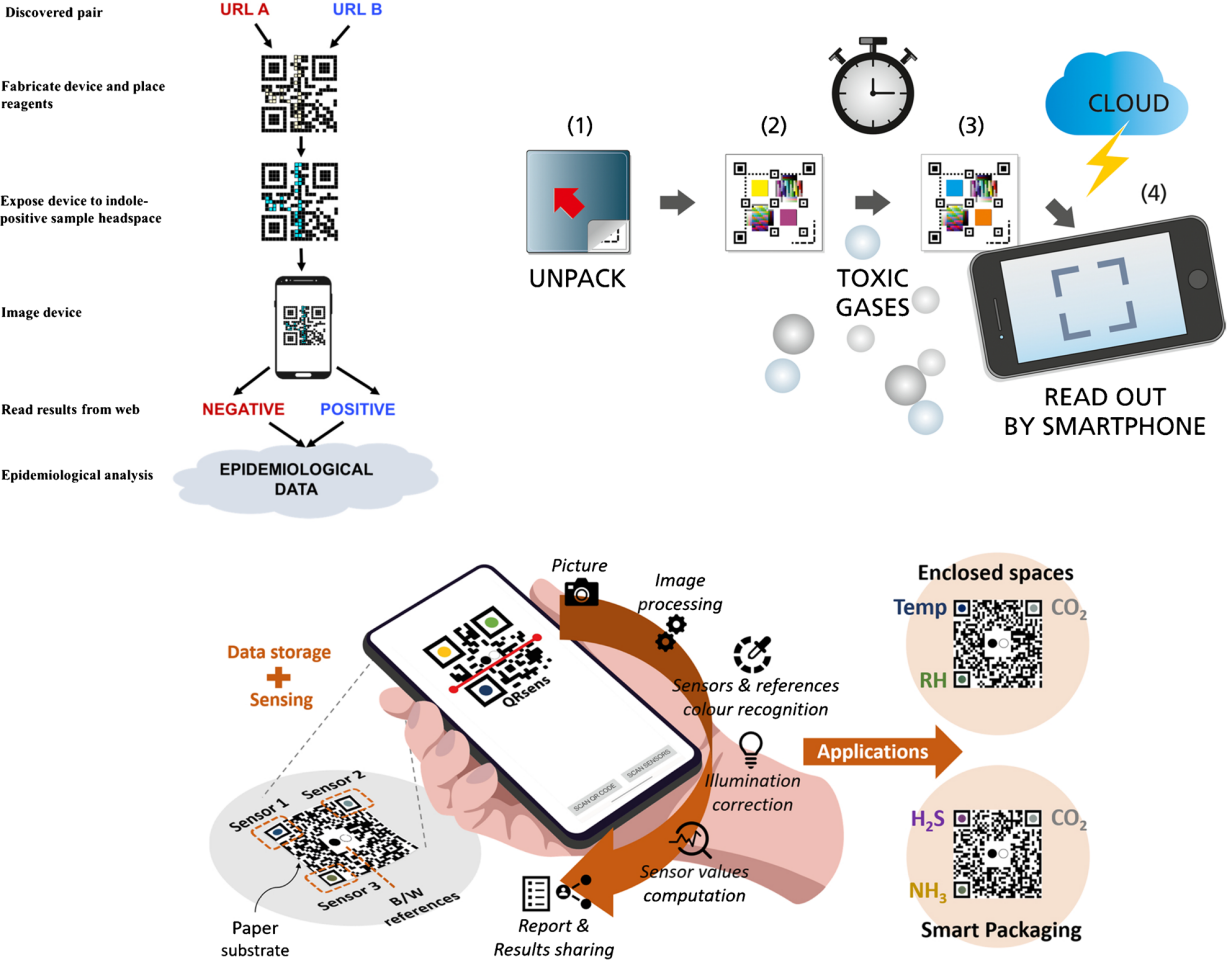
Examples of QR code-like colorimetric devices. Reprinted with permission from [47, 48, 50]

The applicability and convenience of QR code paper-based platforms for the detection of volatiles with colorimetric readout have been recently demonstrated. Modified QR codes can be used for sensing purposes while maintaining their initial functionalities as a data carrier. For instance, a QR code modified in specific reaction regions with the colorimetric reagent p-dimethylaminocinnamaldehyde enabled the detection of the volatile biomarker indole when exposed to the headspace of *E. coli* culture [50]. Very recently, an appealing enhanced QR code, named as QRsens, has been reported [48]. QRsens maintains its original functionality as a data storage device while providing multiple sensing capabilities. QRsens can be used with an ad hoc smartphone application, and the color correction of ambient illumination conditions is automatically conducted. Thus, the proposed QRsens shows much potential for volatile sensing in enclosed spaces, smart packaging, etc. Representative examples of QR code-like colorimetric devices are depicted in Fig. 2. QR codes modified with optically responsive materials have also been exploited for food freshness assessment and for the development of sensor arrays in order to discriminate volatiles [51, 56], selected applications being described in the corresponding subsections below.

Different contributions have focused on cellulose-based sensors with luminescent detection for volatile sensing. For instance, paper discs impregnated with bimetallic europium(III)/copper(II) complex chemosensors have been reported for H_2_S sensing [57]. The luminescence of the lanthanide-based sensor was found to be quenched in the presence of Cu(II), whereas a luminescence switch-on was observed by exposure to H_2_S, with an LOD of 100 ppb in less than 30 s. The sensors showed certain reversibility for up to 6 cycles, even though with progressively reduced sensitivity due to the increasing formation of CuS. Cellulose substrates impregnated with bis(2-aminobenzoic)palladium(II) complex and ethylene glycol as humectant enabled the sensitive fluorimetric detection of H_2_S in the air [58]. The assay was based on the increase in the fluorescence intensity associated to PdS formation with the release of the luminescent ligand.

An ethyl cellulose platform modified with platinum(II) meso-tetrakis(pentafluorophenyl)porphyrin and 1-hydroxy-3,6,8-pyrenetrisulfonic acid trisodium salt as O_2_- and CO_2_-sensitive materials, respectively, along with 7-amino-4-trifluoromethyl coumarin as the reference blue emission dye, enabled the simultaneous detection of O_2_ and CO_2_ by using a single (405 nm) LED as the excitation source and without any spectral overlap [59]. The reversible fluorescence color-shift properties of 2,5-dimethoxy-1,4-bis[2-(4-pyridyl)-ethenyl]benzene enabled the ratiometric detection of CO_2_ (LOD: 5.7 ppm) with a sensor response of 1 min, even though matrix effects were noticeable when applied to exhaled breath analysis [60].

The porosity of cellulose has remarkably been exploited in chemiluminescence for altering the flash-type luminol-H_2_O_2_ system to long-lasting emission. In particular, the immobilization of a Cu(II)-1,10-phenanthroline-2,9-dicarboxylic acid complex (as a catalyst of the system) in the pores of filter paper led to a longer emission time (> 30 min) associated to a slow diffusion rate, unlike in solution or on other non-porous substrates [61]. This strategy has been applied to the determination of H_2_S, based on its reaction with Cu(II).

The hydrophilic nature of cellulose-related materials has been exploited for the development of humidity sensors. For instance, o-MWCNTs have been used as the sensitive material in a paper-based humidity sensor that showed a minimum precision drift of 0.2% relative humidity per month [43]. The response to gaseous water molecules has been attributed to a decrease in conductance through charge transfer between o-MWCNTs and H_2_O. Composites of cellulose nanofibers with dispersed graphene [28], as well as 2,2,6,6-tetramethylpiperidinyl-1-oxyl (TEMPO)-oxidized nanofiber and MWCNTs [41], have also been reported with this aim, showing remarkable stability (15 days). In addition, printable flexible resistive-type humidity sensors fabricated with cellulose nanofibers/carbon black [30] and cellulose nanofibers/graphene nanoplatelet [31] composite inks, as well as a conductive cellulose nanofiber/carbon nanotube foam sensor [62], have demonstrated their applicability to non-contact fingertip moisture and human breathing monitoring.

QCM humidity sensors have also exploited the hydrophilicity of cellulose-based materials for enhanced water adsorption and, therefore, performance. In this sense, nitro-modified cellulose nanocrystals [35] have been reported as responsive films with rapid response and recovery times and a stability of ca. 20 days.

Besides cellulose-based sensors with single readout, a number of recent contributions have dealt with the development of captivating dual-mode detection strategies. For instance, inkjet-printed sensor strips modified with solvatochromic polydiacetylenes have been reported for chloroform identification based on the selective color change from blue to yellow accompanied by green fluorescence [45]. Lead halide perovskite nanostructures grown on the fibers of cellulose substrates display excellent stability (4 months) and allow the detection of ammonia gas with colorimetric and electrical readout based on the decomposition of the corresponding materials to lead halides [63]. In addition, PADs prepared by immobilization of 4-mercaptopyridine-modified gold nanorod-reduced graphene oxide hybrids, anhydrous methanol, and starch-iodine complex enabled a colorimetric and SERS dual-mode assay for determination of released SO_2_ in wine [39]. Particularly, a decrease in the deep blue color of PADs, attributed to the iodine–starch complex, occurred in the presence of SO_2_ due to the Karl-Fisher reaction, whereas an increase in SERS signals occurred due to the conversion of 4-mercaptopyridine to pyridine methyl sulfate on the gold nanorods. In addition, PADs modified with core–shell gold nanorod-quantum dot-embedded MOF structures, stable for ca. 10 weeks, have recently been applied to the determination of the lung cancer biomarker benzaldehyde in exhaled breath with both fluorescence and SERS detection (LODs: 1.2 and 0.1 ppb, respectively) [40]. A Schiff base reaction between benzaldehyde with the amine group of 4-mercaptoaniline modified on gold nanorods was the basis of the dual-mode strategy. Furthermore, PADs modified in situ with a copper MOF have been recently reported for the dual colorimetric/chemiluminescent determination of volatile sulfur compounds (VSCs) in exhaled air, showing an impressive stability of 18 months [64]. When exposed to VSCs, the copper MOF-modified PAD underwent a color change associated to the formation of CuS, which drastically reduced the catalytic effect of Cu-MOF on the luminol-H_2_O_2_ system.

### Intelligent films for food freshness monitoring

**Fig. 3:**
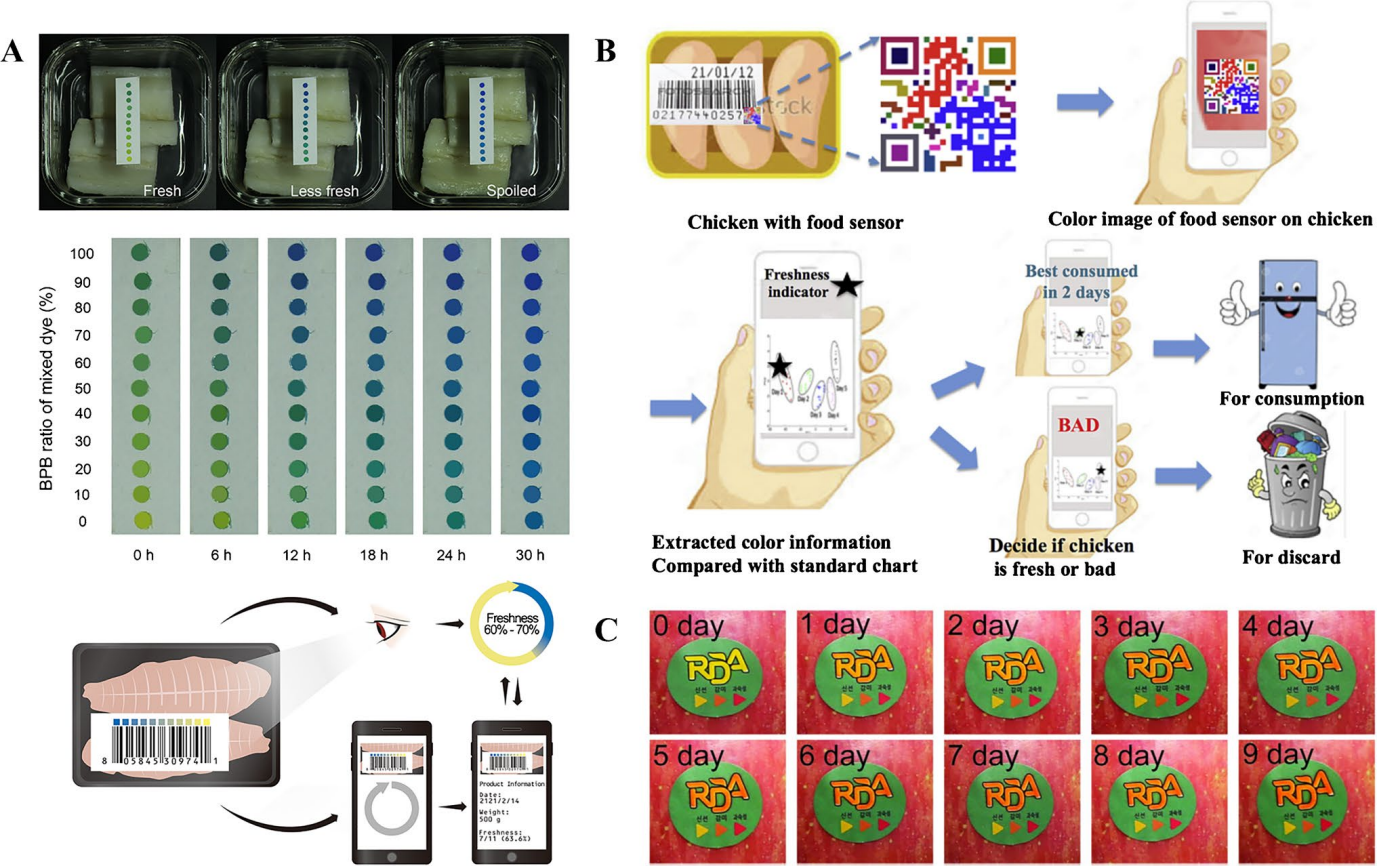
**A** Three main freshness states of the Cod sample. Coloration on the Cod packaging under 25 °C for 30 h. Application outlook of the sensor array. Reprinted with permission from [65]. **B** Schematic diagram of device application as food quality sensor. Reprinted with permission from [56]. **C** Color changes of a sensor label after exposure to apple flavors. The color change to aging (yellow to orange to red) can be observed in the word “RDA,” where the reference colors associated to three stages of apple ripeness (yellow, orange, and red) are displayed in the small triangles below the word “RDA.” Reprinted with permission from [70]

Integration of sensing approaches in food packaging is receiving a great deal of interest to ensure food safety and quality. In this vein, cellulose-based materials modified with a number of responsive materials are demonstrating a high level of suitability for the detection of spoilage. Recent advances in this field are mainly focused on the detection of alkaline and/or acidic volatiles associated to food deterioration (e.g., ammonia, trimethylamine, putrescine). The immobilization of conventional pH indicators in cellulose-based substrates modified toward improved stability is therefore an obvious starting point for real-time food freshness assessment [42]. Novel assays involving pH dyes have been developed as alternative readout strategies, being less dependent on both the subjective perception of single color changes and external lighting conditions. In this sense, a colorimetric strip sensor array resembling a “progress bar” has been recently proposed by mixing different amounts of two pH indicators with the same color-changing types (yellow to blue), namely bromophenol blue and bromocresol green [65]. An increased number of blue spots in the film occurred when exposed to increased amounts of basic volatiles (Fig. 3A), thus allowing a more accurate naked-eye recognition of food quality. Besides, a text-displaying indicator card based on a distance-based color transition has also been reported for the freshness assessment of shrimps [66]. Interestingly, the lamination of the indicator card with the exception of one side part enabled the received vapors to diffuse through the PAD according to a distance-based approach. Ratiometric assays have also been reported. Thus, the pH-dependent oxidation of 3,3’,5,5’-tetramethylbenzidine (TMB) by singlet oxygen, generated in the presence of a photosensitizer (2,4’,5’,7’-tetraiodofluorescein) by a green LED, was the basis for a recently reported paper-based device for spoilage monitoring stable for nearly 1 month, where the color of the photosensitizer served as a background for the analytical measurement [67]. The applicability of sensor arrays integrated in a QR code with colorimetric smartphone readout has also been described. Thus, a cellulose substrate modified with microbeads containing three different dyes sensitive to a number of relevant volatiles has been used for the assessment of chicken aging status (Fig. 3B) [56].

The synthesis of novel pH indicators with potential applicability in intelligent color-changing packaging sensors is also receiving attention. As an example, two pH-sensitive dyes based on the combination of anthraquinone and azo chromophores can be covalently attached to a paper substrate by printing, showing applicability for the freshness assessment of cooked crabs [49]. In addition, pH-responsive poly(amidoamine) dendritic macromolecules functionalized with spiropyran and doped with oxazolidine derivatives have also found applicability as photoluminescent paper indicators for monitoring acidic vapors generated by bacterial activities from spoiled milk [68]. Thus, the protonated form of the chemosensor displayed red fluorescence emission under UV irradiation (365 nm).

Alternatives to synthetic pH indicators include pH-responsive natural dyes such as anthocyanins or curcumin. For instance, sodium carboxymethyl cellulose films containing gelatin/chitosan and curcumin microcapsules have been proposed for quality monitoring of pork using colorimetric smartphone detection [69]. Additionally, anthocyanin-containing films prepared with cellulose acetate and cellulose nanofibers, which can be transformed into biodegradable bags taking advantage of the thermoplastic nature of cellulose acetate, have very recently been proposed for the assessment of fish and meat freshness [18].

Attention has also been paid to H_2_S monitoring in food samples. For instance, a chemiresistive sensor based on tungsten disulfide nanosheet–decorated cellulose nanofiber–templated copper oxide nanocomposites, showing stability of 1 month, has been recently reported for the assessment of eggs freshness [32]. In addition, a thin layer of an optically transparent nanocomposite based on cellulose nanocrystals, AgNPs, and alginate-molybdenum trioxide NPs showed promising sensing properties toward H_2_S, associated to the formation of Ag_2_S and reduction of the Mo oxidation state upon exposure to the volatile [26].

Alternative sensing devices have been reported in the literature for monitoring other volatiles of much relevance in food packaging such as aldehydes, ethylene, or water, with applicability in the monitoring of fruit freshness and ripeness. Thus, sensor labels (Fig. 3C) consisting on the use of pH indicators under a controlled concentration of hydroxide ions enabled the colorimetric detection of aldehydes as markers of fruit ripeness [70] on the basis of the Cannizzaro reaction.

Additionally, a flexible thiol-functionalized liposomal polydiacetylene colorimetric sensor film, fabricated with cellulose nanocrystals and chitosan, has been proposed for the naked-eye monitoring of ethylene [71]. In the presence of the volatile, a Michael addition reaction occurs, leading to a blue-to-red color transition associated to a conformational alteration in the polymers from planar to non-planar.

Multifunctional composite materials that fulfil several desirable features apart from their capability for volatile monitoring are receiving increasing attention. In this sense, the use of Ag-MOFs@carboxymethyl filter paper, with both antimicrobial properties and humidity response, has been recently proposed for preservation and fruit quality monitoring [72]. The multifunctional composite paper showed promise for preservation and remote monitoring purposes during transportation and storage based on the gradual resistance reduction of the composite material associated to the increased water adsorption.

### Discrimination of volatiles: sensor arrays

A number of paper-based optoelectronic noses have been reported in the literature. Sensor arrays, firstly introduced by Suslick et al. [73], involve a carefully selected array of chemoresponsive dyes whose optical properties are modified in the presence of target molecules with subsequent discrimination by pattern recognition methods such as PCA, hierarchical cluster analysis, or linear discriminant analysis, among others. In this seminal work [73], a variety of solid supports were modified with metalloporphyrins, pH indicator dyes, and solvatochromic dyes which respond to changes in the Lewis acidity or basicity, (Brønsted) acidity or basicity, and polarity, respectively, achieving the discrimination of 32 VOCs in accordance with the colorimetric array responses and PCA. Thus, the unique color-difference patterns obtained when the responsive materials were simultaneously exposed to volatiles allowed for their discrimination on the basis of standard chemometric methods. Since then, a wide range of substrate materials has been used for the fabrication of sensor arrays, including impermeable substrates, cellulose-based materials, and porous polymer membranes, among others. The selection of substrate materials shows a paramount role on the performance of sensor arrays [74]. In this respect, cellulose-based materials exhibit an intermediate performance when considering their optical transparency, their compatibility with the responsive materials, the quality of printed spots, response time, and compatibility with potentially high humidity [74, 75]. Further contributions involving cellulose substrates modified with carefully selected responsive materials include, among others, organic molecules (e.g., pH indicators, aniline dyes, a range of solvathochromic dyes, polydiacetylenes) [44, 76, 77]; metalloporphyrins [44]; and NPs (e.g., metallic NPs with different capping ligands and polymeric NPs) [46, 78, 79].

**Fig. 4:**
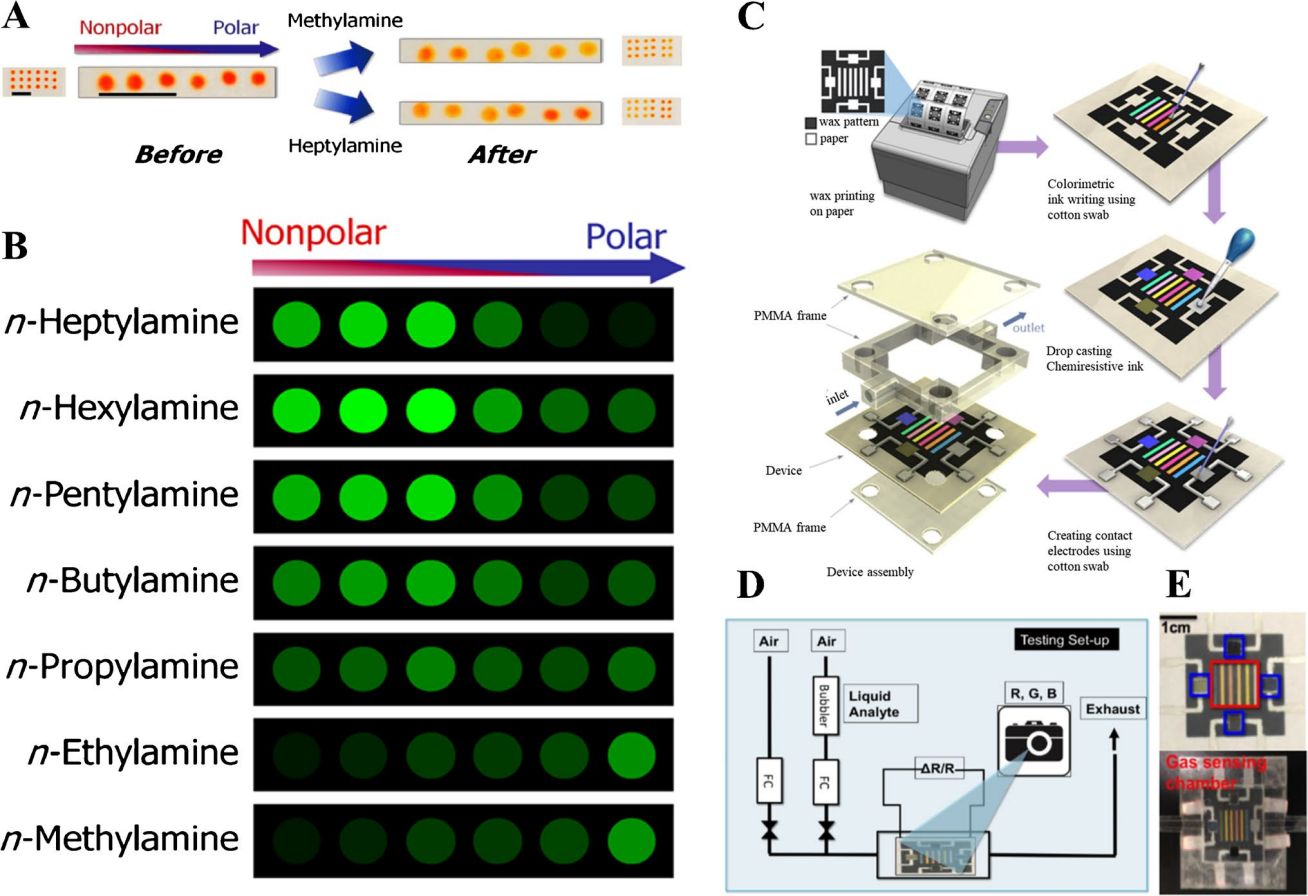
**A** Color scan of a triplicate sensor array. The overall polarity of the spots increases from left to right. **B** Color-difference patterns of sensor arrays after exposure to various amine gases. Reprinted with permission from [46]. **C** Patterning and functionalization of paper-based optoelectronic sensors. **D** Test setup. € Full device. Reprinted with permission from [44]

Different contributions can be highlighted here. Thus, a QR code modified with 12 selected dyes, including pH indicators, metalloporphyrins, solvatochromic dyes, and a specific probe for carbonyls, has been used for olive oil odor identification [51]. Apart from discriminating three different types of oil as well as oxidized and non-oxidized olive oil samples according to their volatile fraction, the PADs also enabled the quantitative determination of nonanaldehyde as an indicator of rancidity. The modification of cellulose substrates with metallic NPs obtained with a range of capping agents has also been reported to enable the discrimination of volatile compounds based on the color change associated to the volatile-induced aggregation of NPs. Thus, hydrophilic substrates modified with Au and Ag NPs of varying surface properties have enabled the discrimination of 45 volatiles [78]. Furthermore, a hydrophobic paper-based sensor array composed of 16 metallic NPs has been reported as a potential screening alternative for early diagnosis of leukemia on the basis of the responses of the sensor array to blood vapors [79]. Apart from metal NPs, sensor arrays fabricated by combining a “class-selective” chromogenic dye (e.g., a derivative of the amine-responsive azo dye ETH^T^ 4001) with polymer NPs of varying polarities have also been reported (Fig. 4A and B), enabling the discrimination of seven volatile amines at concentration levels above 50 ppm in standard copy paper [46].

The combination of colorimetric and chemiresistive sensor arrays in a given device has also been reported with the aim of achieving improved discriminative power when compared with each specific detection approach [44]. The device, fabricated by direct writing of sensing materials on chromatography paper, consisted of six chemoresponsive dyes, including pH indicators, solvatochromic dyes, and a metalloporphyrin, whereas the chemiresistive sensors comprised carbon nanotubes, graphite, conductive polymers, and an ionic liquid (Fig. 4C–E).

Fluorimetric detection has also been reported for the identification of organic volatile compounds. For example, cellulose substrates modified with three benzothiazole-salicylidene derivatives enabled the recognition of 15 VOCs by PCA [52], whereas a paper-based fluorescence array based on 36 sets of soluble conjugated polymeric NPs allowed for detecting 30 hazardous volatile analytes, including explosives [80].

Finally, a paper-based cantilever sensor array enabled the discrimination of four VOCs, namely acetone, methanol, ethanol, and terahydrofuran [81]. The sensor unit, fabricated with eight different swellable polymers selected in accordance with the “like dissolves like” principle, selectively responded to each VOC with an angle pattern measurable by a printed protractor facing the cantilevers.

## Cellulose-based sensing of volatile derivatives

In situ formation of volatile derivatives has been exploited to expand the applicability of PADs and microfluidic PADs for the determination of ionic species of analytical relevance. Selected applications are discussed in the sections below and detailed information is summarized in Table 3.

**Table 3:** Selected applications of PADs and microfluidic PADs for detection of volatile derivatives

Analytes	Recognition element	Cellulose material	Detection	LOD	Repeatability (RSD, %)	Samples analyzed	Analysis time	References
*PADs*								
Arsenic(III) and total arsenic	Ag(I)	Whatman no. 3 filter paper	Colorimetric (scanner)	1.1 µg/L	7.1%	Water	5 min	[82]
Nitrite and sulfide	Griess reagent/Cu(II)	Whatman 602H filter paper	Colorimetric (scanner)	55 and 5 µg/L	5.9% and 6.7%	Water	15 min	[83]
Bromide and bromate	Silver nanoprisms	Whatman no. 1 filter paper	Colorimetric (distance)	10 and 0.5 µg/L	3.29–11–10% and 2.23–14.34%	Water, rice, and flour	40 min	[84]
Cyanide	Au@AgNPs	Whatman no. 1 filter paper	Colorimetric (distance)	10 µg/L	5.38–7.20%	Wastewater	40 min	[85]
Selenium	CdTe QDs	Chromatography paper	Fluorimetric (visual detection)	0.1 µg/L	2.4%	Urine	10 min	[86]
Zinc	Au nanoclusters	Chromatography paper	Fluorimetric (visual detection)	3 µg/L	2%	Whole blood and cells	15 min	[87]
Iodide	Cu nanoclusters	Whatman no. 1 filter paper	Fluorimetric (smartphone)	29.0 µg/L	–	–	15 min	[88]
Ammonium	Citric acid/cysteine fluorophore	Whatman no. 1 filter paper	Fluorimetric	37 µM	5.8%	Water and sediments	25 min	[90]
Bromide and bromate	Citric acid/cysteamine fluorophore	Whatman no. 1 filter paper	Fluorimetric (smartphone)	5.4 and 0.9 µg/L	8.3 and 6.3%	Water	30 min	[91]
Nickel(II)	CdTe QDs	Chromatography paper	Fluorimetric (smartphone)	6.2 µg/L	3.2%	Tea infusion	20 min	[92]
Arsenic, antimony, and bismuth	AgNPs	Whatman 602H filter paper	ICP-MS	1–15 ng/L	1.2–2.7%	Water	10 min	[93]
Selenium(IV)	AuNPs	Whatman grade 44	Colorimetric (scanner)	12 µg/L	4–5%	Water	3 min	[94]
Sulfide	Au@AgNPs	Whatman 1PS	Colorimetric (smartphone)	14.7 µg/L	4.4%	Water	22 min	[95]
*Microfluidic PADs*								
Ammonium	3-nitrophenol, bromothymol blue	Whatman grade 4 filter paper	Colorimetric (scanner)	0.8 and 1.8 mg/L (as N)	3.1% and 3.7%	Sewage and soil water	2–6 min	[96]
Ammonium	Nessler reagent and 3-nitrophenol	Whatman grade 4 filter paper	Colorimetric (digital camera)	3.14 and 8.99 mg/L (as N)	1.08% and 0.97%	Wastewater and fertilizer	5–6 min	[99]
Ammonium	Red rose extract (anthocyanins)	Whatman grade 1 filter paper	Colorimetric (digital camera)	2.25 mg/L (as N)	2.28%	Fish pond water	1.5 min	[100]
Total ammonia	Nitrazine yellow and bromothymol blue	Whatman grade 4 filter paper	Colorimetric (scanner)	0.32 and 0.47 mg/L (as N)	2.5% and 9.0%	Freshwater	5 min	[103]
Sulfide	DMPD			–	–	–	2 min	
Ethanol	K_2_Cr_2_O_7_			–	–	–	3 min	
Arsenic(III) and total arsenic	Au(III)	Whatman grade 4 filter paper	Colorimetric (scanner)	0.41 and 0.43 mg/L	6.74% and 6.91%	Groundwater and freshwater	5 min	[98]
Mercury(II)	Starch	Whatman grade 4 filter paper	Colorimetric (digital camera)	20 mg/L	2.2%	Contaminated soil and water	9 min	[101]
Iodate	Albumin-stabilized gold nanoclusters	Filter paper no. 2	Fluorescence (fluorimetry and smartphone)	5 µM and 10 µM	< 3%	Iodized salt and fish sauces	15 min	[102]
Sulfite	Sea urchin-like ZnO NPs	Chromatography paper	SERS	2 mg/L	4.17%	Wine	5 min	[97]
Sulfite	H_2_O	Whatman grade 4 filter paper	C4D	6.61 mg/L	0.9–5.0	Wine and juice	3.3 min	[104]
Sulfite	Graphene electrode	Filter paper	SWV	1.5 mg/L	1.12%	Wine	8 min	[105]
Nitrogen oxides	CuNPs/screen-printed graphene electrode	Whatman no. 1	DPV	0.23 ppm 0.03 ppm	< 5.1%	Indoor and outdoor air, exhaust gases from cars	25 min 1 h	[106]

*C4D*, capacity coupled contactless conductivity detector; *DMPD*, N,N-dimethyl-*p*-phenylenediamine; *DPV*, differential pulse voltammetry; *SWV*, square wave voltammetry

### PADs for determination of ionic species

Sensing approaches involving cellulose-based sensors can be extended to the determination of non-volatile analytes via in situ formation of appropriate volatile derivatives. This option enables the determination of target analytes in condensed matrices with improved selectivity when appropriate responsive materials and selective derivatization reactions are used. Furthermore, excellent sensitivity can be achieved due to the preconcentration involved in the process. For instance, a careful selection of experimental conditions for in situ formation of AsH_3_ has enabled the colorimetric determination of As(III) and total inorganic As in waters at concentration levels significantly lower than the maximum contaminant level set by the USEPA (10 ng/mL) by means of PADs modified with a Ag(I) salt in its reaction area [82]. The formation of hydrophobic barriers in the PAD avoided potential reactions between the recognition element and the derivatization reagent. The selection of common derivatization conditions and selective recognition elements in separate detection areas of the PAD has also been exploited for the simultaneous determination of non-volatile analytes with in situ formation of volatiles [83].

An alternative non-instrumental distance-based detection assay involving silver nanoprisms as the recognition element has been proposed for the determination of inorganic bromine species in water and food samples [84]. The assay for bromate determination involved enrichment of in situ formed Br_2_ (by a circular paper substrate) and elution prior to the analysis, whereas a direct assay was devised for bromide determination. A color change from pink to yellow, attributed to oxidative etching of silver nanoprisms in the channels of the PAD, enabled the quantitative determination of bromine species within 40 min. This concept has also been exploited for cyanide determination in water samples via in situ HCN generation and trapping of the volatile by a paper disc impregnated with 0.1 M NaOH solution, with subsequent distance-based detection using core-shell Au@AgNPs as recognizing element [85]. The detection of the anion is based on measuring the colorless length associated to etching of (yellow) Au@AgNPs.

On the other hand, the combination of three-phase microseparation approaches with non-conventional fluorimetric detection has also been assessed. Thus, PADs modified with CdTe quantum dots [86] and gold nanoclusters [87] have enabled the sensitive determination of selenium [86] and zinc [87] in biological samples, respectively, on the basis of in situ hydride generation and fluorescence quenching of the corresponding nanoprobes. Additionally, the luminescence quenching of polyvinylpyrrolidone-capped copper nanoclusters in presence of I_2_ has been exploited for the determination of iodide in waters [88]. Furthermore, PADs modified with citric acid-derived fluorophores, which have been identified as the main responsible of the luminescence of certain carbon dots [89], have been exploited for the solid-state luminescent determination of ammonia in waters and extractable ammonia in marine sediments on the basis of a pH-dependent dual excitation/dual emission ratiometric approach [90]. In addition, bromine speciation in environmental waters has been performed on the basis of the irreversible formation of a non-luminescent dibrominated derivative of the probe in the detection area of the PAD when exposed to in situ generated Br_2_ [91].

**Fig. 5:**
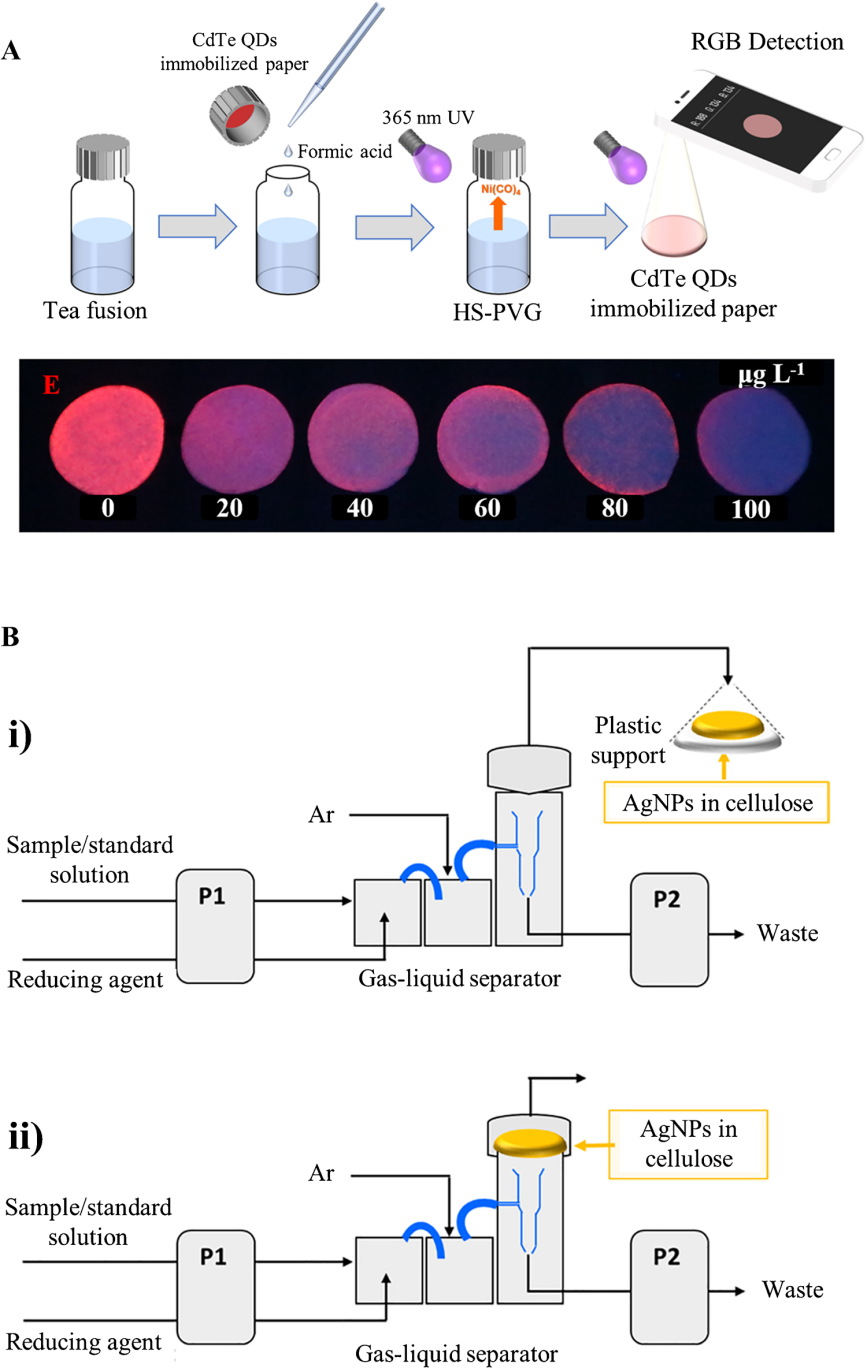
**A** Schematic diagram of the experimental setup of the photochemical vapor generation-headspace thin-film microextraction method for Ni(II) determination. Adapted with permission from [92]. **B** Experimental setups used for continuous hydride generation and trapping onto AgNP-modified cellulose filters. Adapted with permission from [93]

The above methods required chemical derivatization reactions for a volatile generation. Alternatively, photochemical reactions can be implemented for generating volatile derivatives, even though this option has not been explored sufficiently. In this vein, the applicability of photochemical vapor generation in headspace thin-film microextraction has been recently demonstrated for the smartphone-based luminescent detection of Ni(II), a non-hydride-forming element (Fig. 5A) [92]. In the presence of formic acid, Ni was photochemically converted into volatile Ni(CO)_4_, the volatile being subsequently converted into NiO in the headspace, which led to the fluorescence quenching of CdTe quantum dots immobilized in a cellulose substrate.

Alternatives to batch headspace methods for the enrichment of volatiles in cellulose substrates have been recently proposed. In particular, cellulose-based platforms modified with AgNPs enabled effective retention of volatile hydrides based on the catalytic decomposition of the volatiles in the substrate [93]. Two configurations for hydride trapping were attempted (Fig. 5B), with the modified cellulose substrate enclosed inside a conical polypropylene chamber or integrated into the gas-liquid separator unit, the second approach leading to improved retention of the volatiles.

Cellulose substrates have also been applied for detection purposes in combination with liquid-phase microextraction. The analysis, however, usually required spotting the enriched microdrop on a hydrophilic paper substrate [94]. In a recent contribution, we have demonstrated the applicability of waterproof cellulose–based substrates as holders for carrying out in-drop enrichment and smartphone-assisted plasmonic colorimetric detection without the requirement of transferring the enriched microdrop to a sample compartment for analysis [95].

### Microfluidic PADs for determination of ionic species

**Fig. 6:**
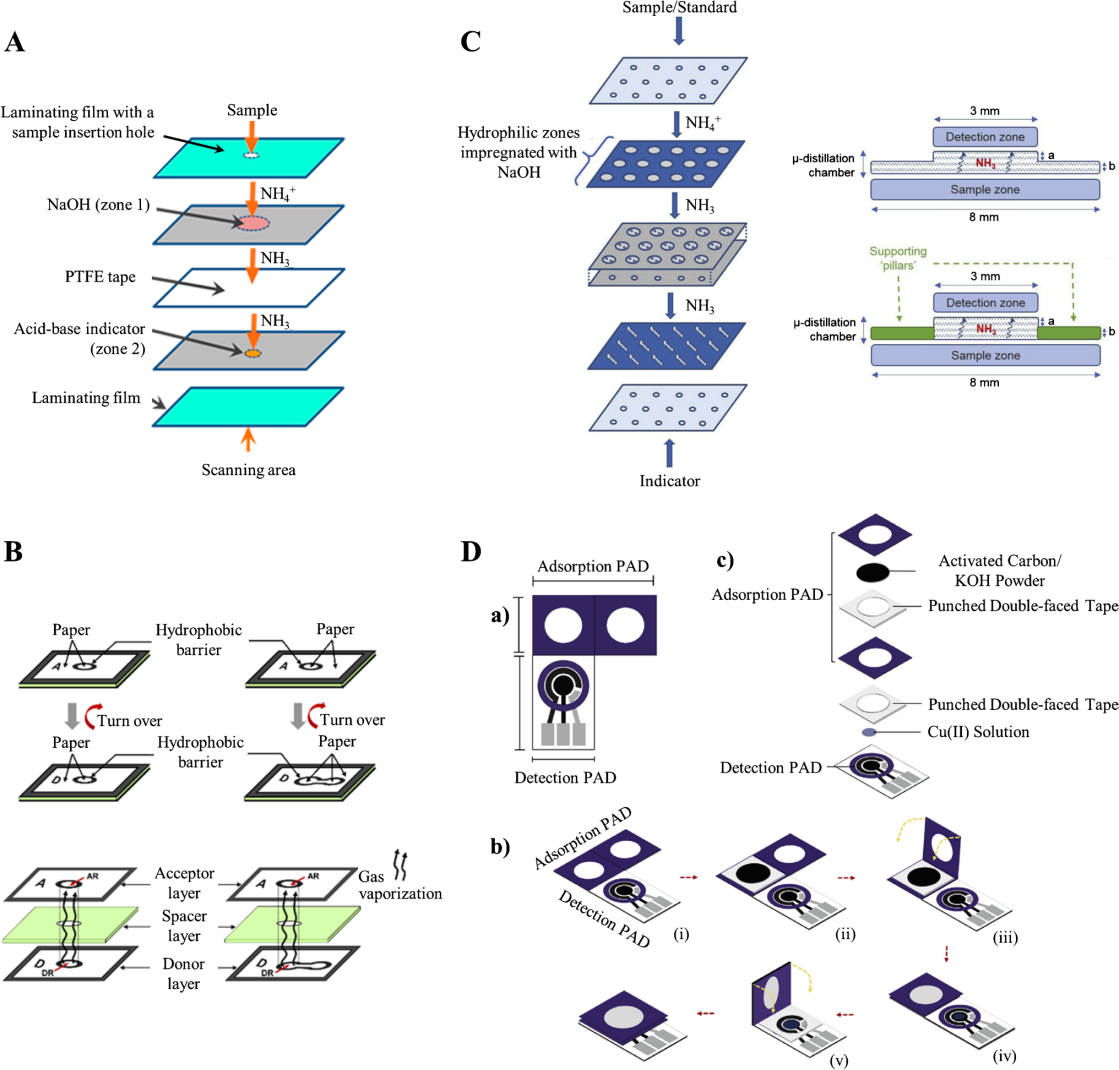
**A** Schematic diagram of the gas-diffusion μPAD. Adapted with permission from [96]. **B** Schematic diagrams of two devices for analysis of volatile and non-volatile compounds by membraneless gas-separation μPADs. Adapted with permission from [99]. **C** Schematic representation of the μPAD design. Adapted with permission from [103]. **D** Fabrication of the gas sensing-PAD; (a) design of the origami device (blue zones indicate wax-impregnated areas); (b) fabrication started with cutting out the paper and printing (i), followed by attachment of the punched double-sided adhesive tape and filling with carbon (ii) and accomplishment of the adsorber by folding the paper (iii, iv); finally, the adsorber was fixed to the sensing unit (v); (c) exploded view of the sensor. Adapted with permission from [106]

µPADs represent miniaturized, portable, and affordable platforms for the determination of ionic species via in situ volatile generation. Remarkable improvements, especially related to microfluidic design, have been recently achieved. Kolev et al. [96] implemented a membrane-based gas-diffusion separation approach on a µPAD for the colorimetric determination of molecular ammonia and ammonium ions in waste and soil waters (Fig. 6A). The assay involved the quantitative conversion of NH_4_^+^ to NH_3_, diffusion of the volatile across a hydrophobic microporous Teflon membrane, and subsequent reaction with an acid-base indicator in the corresponding layer. The gas-diffusion approach has been exploited for the development of alternative assays in order to determine volatile analytes or derivatives, including sulfites and inorganic arsenic species by the formation of SO_2_ [97] and AsH_3_ [98], respectively. Thus, sea urchin-like ZnO NPs grown on paper, which display a shelf lifetime of 16–60 days depending on the storage conditions, have enabled the determination of sulfite in wines with SERS detection [97]. In situ formation of AuNPs in the detection area of the µPAD enabled the scanometric determination of As(III) and total As in waters [98], even though with LODs (0.41 mg/L and 0.43 mg/L, respectively) well above the concentration levels recommended to prevent potential health problems.

Alternatively, Nacapricha et al. [99] proposed the use of membraneless gas-separation µPADs with two different designs for the determination of volatiles and non-volatile analytes that required in situ volatile generation. The approach consisted of three layers, where a two-sided mounting tape with a circular cut behaving as a spacer layer was sandwiched between the donor and acceptor layers (Fig. 6B). The spacing layer, whose thickness showed an important effect on the analytical response, enabled the volatile analytes or derivatives to diffuse from the donor to the acceptor layer thus leading to the required colorimetric reaction for quantitative analysis. The applicability of the proposed device was demonstrated by the determination of ammonium ions in wastewaters and fertilizers, showing statistically comparable results with those obtained by a reference gas-diffusion flow injection method. Ammonia was also determined in fish pond water samples using a µPAD with the acceptor layer modified with anthocyanins (extracted from a red rose) that shows remarkable stability (at least 1 month) [100]. The proposed approach was also applied to the indirect colorimetric determination of mercury in highly contaminated soils (LOD: 20 mg/L) [101] and the fluorimetric determination of iodate in iodized salts and fish sauces using albumin-stabilized gold nanoclusters as a recognition element [102]. In situ iodine generation was carried out in both contributions.

More recently, a microdistillation chamber and inclusion of supporting pillars resulted in an enhanced sensitivity as compared to previously reported gas-permeable membrane-based and membraneless-based µPADs (Fig. 6C) [103]. A number of separate layers of modified paper substrates are typically used in these approaches, thus requiring lamination of the µPADs to maintain the alignment of donor/acceptor areas while minimizing vapor leakages.

A number of remarkable contributions involving µPADs with alternative detection systems, including conductivity and voltammetric detection, have also been reported. A membraneless gas separation with in situ generation of SO_2_ has been reported for the determination of sulfite by monitoring the conductivity change of the moist detection zone (LOD: 6.6 mg/L) [104]. In addition, a paper-based three-layer microfluidic lid has been designed as a key component of a microchamber device for the determination of sulfite in wines by square wave voltammetry, providing an LOD of 1.5 mg/L [105]. The proposed microfluidic lid enabled a reproducible dispense of the acid reagent needed for generating SO_2_ in situ in a cylindrical acrylic microwell reactor, thus leading to SO_2_ diffusion though a PTFE membrane attached to a graphene electrode. In addition, a disposable µPAD containing activated carbon as an adsorbent for NO and NO_2_ collection and CuNP-modified screen-printed graphene electrode (Fig. 6D) enabled the determination of nitrogen oxides (NOx) in air and exhaust gases from cars by differential pulse voltammetry [106]. The sensor, fabricated in accordance with the principles of origami, showed an LOD below the maximum allowable NOx-levels in ambient air established by the USEPA when using an exposure time of 1 h.

## Conclusions and outlook

The unique properties of cellulose-related materials make them particularly interesting for the development of gas sensors. The latest advances and applicability of cellulose-based sensors to the determination of volatile analytes and derivatives are illustrated throughout the review. As discussed above, a wide range of cellulose-related materials can be exploited for sensing purposes, even though commercially available filters and chromatography papers are widely used. Alternative underexplored (nano)cellulose–based materials might have more appropriate properties for certain applications in the field of volatile detection and they should therefore be evaluated more profusely. The application of more efficient fabrication methods is also required to significantly reduce the number of responsive materials needed to prepare modified substrates with sensing capabilities while alleviating potential reproducibility issues. The opportunities opened up by the outstanding contributions developed in the field of material sciences are countless. In this vein, the implementation of novel materials with remarkable physicochemical properties in cellulose substrates can be of much interest for volatile sensing. The advances achieved toward the development of multipurpose materials involving cellulose-related materials for non-destructive sensing among other aims are also remarkable, e.g., in the intelligent packaging industry, and further contributions are expected to occur in other areas.

Additional aspects such as the stability and reusability of cellulose-based sensors are of paramount importance when considering their application and potential commercialization.

The search for expeditious and straightforward analytical approaches with enhanced sensitivity and, more importantly, selectivity, is needed to expand even more the scope of applications of paper-based substrates for the detection of trace and ultratrace volatile analytes or derivatives. In particular, sensitivity improvements can be achieved by considering previously reported strategies for offline and online enrichment and/or detection approaches, even though further efforts are still particularly required in µPADs. The significance of recent developments in this area is beyond doubt but, despite this, additional design improvements could partially alleviate the reduced sensitivity associated mainly to very small sample volumes.

Last but not least, there is much room for improvement toward more environmentally friendly fabrication and sensing strategies. Increasing attention should be paid to the environmental, health, and safety issues associated to the chemicals and solvents used for the fabrication of paper-based substrates for volatile sensing. The miniaturization of analytical systems through the implementation of paper-based devices may suggest that the consumption of reagents and solvents required for their preparation is virtually negligible. However, this is far from being true in a significant number of cases. This is applicable not only to the fabrication of PADs itself, but also importantly to the fabrication of cellulose-related substrates and the synthesis of the responsive materials immobilized on them. Identifying such limitations can contribute to a continuous improvement of cellulose-based sensors in terms of both analytical performance and sustainability.
